# Bis[(1-ammonio­ethane-1,1-di­yl)diphospho­nato-κ^2^ *O*,*O*′]diaqua­nickel(II) nona­hydrate

**DOI:** 10.1107/S160053681001531X

**Published:** 2010-04-30

**Authors:** Vladimir V. Bon, Anatolij V. Dudko, Alexandra N. Kozachkova, Vasily I. Pekhnyo, Natalia V. Tsaryk

**Affiliations:** a Institute of General and Inorganic Chemistry, NAS Ukraine, Kyiv, prosp. Palladina 32/34, 03680, Ukraine

## Abstract

The title compound, [Ni(C_2_H_8_NO_6_P_2_)_2_(H_2_O)_2_]·9H_2_O, exhibits a slightly distorted octa­hedral coordination environment around the Ni^II^ atom. It contains two mol­ecules of 1-amino­­ethyl­idenediphospho­nic acid in the zwitterionic form, coord­inated *via* O atoms from two phospho­nate groups and creating two six-membered chelate rings. Two water mol­ecules in *cis* positions complete the coordination environment of the Ni^II^ atom. The title compound contains nine partly disordered solvent water mol­ecules, which create a three-dimensional network of strong O—H⋯O and N—H⋯O hydrogen bonds.

## Related literature

For general background to the use of organic diphospho­nic acids, see: Matczak-Jon & Videnova-Adrabinska (2005 ▶). For applications of transition-metal bis­phospho­nates, see: Eberhardt *et al.* (2005 ▶). For related structures, see: Li *et al.* (2007 ▶); Dudko *et al.* (2009 ▶).
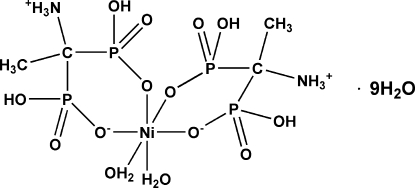


## Experimental

### 

#### Crystal data

[Ni(C_2_H_8_NO_6_P_2_)_2_(H_2_O)_2_]·9H_2_O
*M* *_r_* = 664.95
Monoclinic, 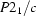

*a* = 15.1408 (3) Å
*b* = 13.1972 (3) Å
*c* = 12.9344 (3) Å
β = 106.1689 (11)°
*V* = 2482.27 (9) Å^3^
*Z* = 4
Mo *K*α radiation
μ = 1.14 mm^−1^
*T* = 173 K
0.23 × 0.22 × 0.15 mm

#### Data collection

Bruker APEXII CCD diffractometer
Absorption correction: numerical (*SADABS*; Bruker, 2005 ▶) *T*_min_ = 0.778, *T*_max_ = 0.850
48425 measured reflections
6235 independent reflections
5333 reflections with *I* > 2σ(*I*)
*R*_int_ = 0.033

#### Refinement

*R*[*F* ^2^ > 2σ(*F* ^2^)] = 0.028
*wR*(*F* ^2^) = 0.072
*S* = 1.07
6235 reflections
415 parameters
H atoms treated by a mixture of independent and constrained refinement
Δρ_max_ = 0.50 e Å^−3^
Δρ_min_ = −0.37 e Å^−3^

### 

Data collection: *APEX2* (Bruker, 2005 ▶); cell refinement: *SAINT* (Bruker, 2005 ▶); data reduction: *SAINT*; program(s) used to solve structure: *SHELXS97* (Sheldrick, 2008 ▶); program(s) used to refine structure: *SHELXL97* (Sheldrick, 2008 ▶); molecular graphics: *SHELXTL* (Sheldrick, 2008 ▶); software used to prepare material for publication: *publCIF* (Westrip, 2010 ▶).

## Supplementary Material

Crystal structure: contains datablocks I, global. DOI: 10.1107/S160053681001531X/ez2208sup1.cif

Structure factors: contains datablocks I. DOI: 10.1107/S160053681001531X/ez2208Isup2.hkl

Additional supplementary materials:  crystallographic information; 3D view; checkCIF report

## Figures and Tables

**Table 1:** Hydrogen-bond geometry (Å, °)

*D*—H⋯*A*	*D*—H	H⋯*A*	*D*⋯*A*	*D*—H⋯*A*
N1—H1*N*⋯O15^i^	0.82 (2)	2.01 (2)	2.807 (2)	166 (2)
N1—H2*N*⋯O7	0.83 (2)	1.95 (2)	2.773 (2)	172 (2)
N1—H3*N*⋯O17	0.86 (2)	2.00 (2)	2.843 (2)	169 (2)
N2—H4*N*⋯O16	0.90 (2)	1.91 (2)	2.774 (2)	160 (2)
N2—H5*N*⋯O4	0.87 (2)	2.10 (3)	2.955 (2)	169 (2)
N2—H6*N*⋯O23*A*	0.87 (2)	1.98 (2)	2.789 (3)	154 (2)
O3—H3*O*⋯O6^ii^	0.76 (2)	1.81 (2)	2.5637 (18)	172 (2)
O5—H5*O*⋯O2^i^	0.69 (2)	1.91 (2)	2.5930 (18)	170 (3)
O8—H8*O*⋯O11^iii^	0.77 (2)	1.74 (2)	2.5075 (18)	176 (3)
O12—H12*O*⋯O9^iv^	0.73 (2)	1.79 (2)	2.5209 (18)	172 (3)
O13—H131⋯O6^ii^	0.88 (3)	1.83 (3)	2.696 (2)	167 (2)
O13—H132⋯O20	0.72 (2)	1.98 (3)	2.678 (2)	162 (3)
O14—H141⋯O9^iv^	0.73 (2)	1.96 (3)	2.6936 (19)	176 (3)
O14—H142⋯O18	0.81 (2)	1.93 (2)	2.711 (2)	162 (2)
O15—H151⋯O12^iv^	0.73 (3)	2.40 (3)	3.029 (2)	145 (2)
O15—H152⋯O1	0.84 (3)	1.96 (3)	2.797 (2)	174 (2)
O16—H161⋯O15^i^	0.88 (3)	1.90 (3)	2.775 (2)	172 (2)
O16—H162⋯O17^i^	0.78 (3)	2.06 (3)	2.838 (2)	177 (3)
O17—H171⋯O18^i^	0.81 (3)	2.03 (3)	2.835 (2)	170 (3)
O17—H172⋯O21	0.88 (3)	1.96 (3)	2.786 (2)	155 (2)
O18—H181⋯O22*A*^v^	0.95 (3)	1.79 (3)	2.702 (2)	158 (2)
O18—H182⋯O11^vi^	0.83 (3)	2.02 (3)	2.841 (2)	168 (2)
O19—H191⋯O2	0.81 (3)	1.98 (3)	2.781 (2)	169 (3)
O19—H192⋯O13^vii^	0.85 (3)	2.23 (3)	3.055 (2)	163 (2)
O20—H201⋯O21^viii^	0.94 (3)	1.91 (3)	2.829 (3)	164 (3)
O20—H202⋯O22*A*^v^	0.90 (3)	2.05 (3)	2.861 (4)	149 (3)
O21—H211⋯O19	0.85 (3)	1.99 (3)	2.814 (2)	163 (3)
O21—H212⋯O19^ix^	0.88 (3)	2.06 (3)	2.928 (3)	166 (3)
O22*A*—H221⋯O3	0.94 (3)	1.86 (3)	2.775 (2)	167 (3)
O22*A*—H222⋯O10^ii^	0.89 (3)	2.10 (3)	2.958 (3)	161 (2)
O23*A*—H231⋯O16^x^	0.76 (3)	2.62 (3)	3.225 (4)	139 (3)
O23*A*—H232⋯O20^xi^	0.89 (3)	2.14 (3)	2.951 (4)	150 (3)

Symmetry codes: (i) 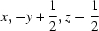; (ii) 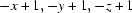; (iii) 
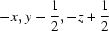
; (iv) 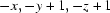; (v) 
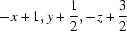
; (vi) 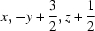; (vii) 
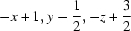
; (viii) 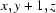; (ix) 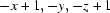; (x) 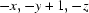; (xi) 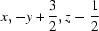.

**Fig. 1.**
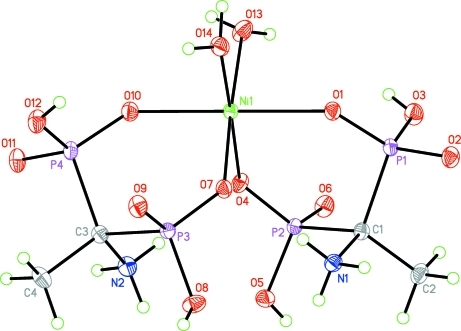
The molecular structure of the title compound showing the atom labelling scheme and 50% probability displacement ellipsoids for the non-hydrogen atoms. Solvent water molecules are omitted for clarity.

**Fig. 2.**
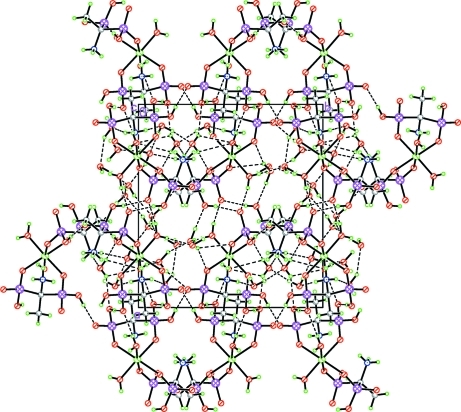
Crystal packing of the title compound, projection along c axis. Dashed lines indicate hydrogen bonds.

## supplementary crystallographic information

### Comment

Organic diphosphonic acids are potentially very powerful chelating agents used in metal extractions and are tested by the pharmaceutical industry for use as efficient drugs for preventing calcification and inhibiting bone resorption (Matczak-Jon & Videnova-Adrabinska, 2005). There is evidence that application of transition metal bisphosphonates can improve fixation of cementless metal implants by enhancing the extent of osseointegration (Eberhardt *et al.*, 2005). In this respect a detailed structure-correlated study of the individual properties and the complex-forming driving factors is desired in order to sufficiently understand bisphosphonate physiological activity.

Several structures of Ni^II^ and Zn(II) aminoethylidenediphosphonates have been reported previously (Li *et al.* 2007). The main difference between these and the title compound is the presence of two water molecules instead of 1,10-phenanthroline in the coordination environment of the transition metal ion (Li *et al.* 2007).

The asymmetric unit of the title compound contains one molecule of the complex (Fig. 1). Two molecules of 1-aminoethylidenediphosphonic acid chelate the central metal ion via two oxygen atoms from phosphonic groups forming six membered non-planar metallocycles. Two water molecules situated in *cis-*positions complete the slightly distorted octahedral coordination environment of the Ni^II^. The Ni—O bond lengths have expected values, which correlate with previously reported related structures (Li *et al.*, 2007). The values of the contiguous O—Ni—O angles are in the range of 88.83 (6)° to 92.51 (5)°. The Ni1/O1/P1/C1/P2/O4 and Ni1/O7/P3/C3/P4/O10 metallocycles have envelope conformations with the C1 and C3 atoms 0.8544 (17) Å and 0.7957 (17) Å out of planes, respectively. The dihedral angle between planar fragments Ni1/O1/P1/P2/O4 and Ni1/O7/P3/P4/O10 equals 85.03 (3)°. The coordinated ligands exist in zwitterionic form with proton transfer from one of the phosphonic groups to the amino group, as found for all 1-aminodiphosphonic acids where the amino group does not participate in coordination (Dudko *et al.*, 2009).

The crystal structure of the title compound contains nine solvent water molecules, which interact with the two coordinated water molecules and the hydrophilic phosphonic groups. As a result, a 3-D network of mostly strong O—H···O and N—H···O H-bonds has been found in the structure (Fig. 2; Table 1).

### Experimental

Light green crystals of the title compound were obtained from a mixture of 10 ml (10 ^-2^ mol/l) of a water solution of Ni(NO_3_)_2_ with a 20 ml (10 ^-2^ mol/l) solution of 1-aminoethylidenediphosphonic acid. The resultant solution was stored in a dark place for slow evaporation. After the 20 days suitable crystals for X-ray data collection were obtained.

### Refinement

The refinement of the structure showed two disordered water molecules. O atoms O22 and O23 were split over two sites with occupancies 0.86/0.14 and 0.81/0.19 respectively. The positions with smaller occupancies were both refined isotropically. Hydrogen atoms were found from difference map only for sites with greater occupancy of disordered atom. H atoms bonded to N and O were located in a difference map and refined with Uiso(H) = 1.5Ueq(N) and Uiso(H) = 1.2Ueq(O) respectively. Methyl hydrogens were geometrically constrained and refined using a riding model with C—H = 0.98 Å for CH_3_ [Uiso(H) = 1.5Ueq(C)].

### Crystal data

**Table:**

[Ni(C_2_H_8_NO_6_P_2_)_2_(H_2_O)_2_]·9H_2_O	*F*(000) = 1392
*M**_r_* = 664.95	*D*_x_ = 1.779 Mg m^−^^3^
Monoclinic, *P*2_1_/*c*	Mo *K*α radiation, λ = 0.71073 Å
Hall symbol: -P 2ybc	Cell parameters from 9144 reflections
*a* = 15.1408 (3) Å	θ = 2.3–28.4°
*b* = 13.1972 (3) Å	µ = 1.14 mm^−^^1^
*c* = 12.9344 (3) Å	*T* = 173 K
β = 106.1689 (11)°	Block, light green
*V* = 2482.27 (9) Å^3^	0.23 × 0.22 × 0.15 mm
*Z* = 4	

### Data collection

**Table:**

Bruker APEXII CCD diffractometer	6235 independent reflections
Radiation source: fine-focus sealed tube	5333 reflections with *I* > 2σ(*I*)
graphite	*R*_int_ = 0.033
Detector resolution: 8.33 pixels mm^-1^	θ_max_ = 28.4°, θ_min_ = 1.4°
φ and ω scans	*h* = −17→20
Absorption correction: numerical (*SADABS*; Bruker, 2005)	*k* = −15→17
*T*_min_ = 0.778, *T*_max_ = 0.850	*l* = −17→17
48425 measured reflections	

### Refinement

**Table:**

Refinement on *F*^2^	Primary atom site location: structure-invariant direct methods
Least-squares matrix: full	Secondary atom site location: difference Fourier map
*R*[*F*^2^ > 2σ(*F*^2^)] = 0.028	Hydrogen site location: inferred from neighbouring sites
*wR*(*F*^2^) = 0.072	H atoms treated by a mixture of independent and constrained refinement
*S* = 1.07	*w* = 1/[σ^2^(*F*_o_^2^) + (0.0345*P*)^2^ + 1.5506*P*] where *P* = (*F*_o_^2^ + 2*F*_c_^2^)/3
6235 reflections	(Δ/σ)_max_ = 0.001
415 parameters	Δρ_max_ = 0.50 e Å^−^^3^
0 restraints	Δρ_min_ = −0.37 e Å^−^^3^

### Special details

**Table:**

Geometry. All esds (except the esd in the dihedral angle between two l.s. planes) are estimated using the full covariance matrix. The cell esds are taken into account individually in the estimation of esds in distances, angles and torsion angles; correlations between esds in cell parameters are only used when they are defined by crystal symmetry. An approximate (isotropic) treatment of cell esds is used for estimating esds involving l.s. planes.
Refinement. Refinement of *F*^2^ against ALL reflections. The weighted *R*-factor wR and goodness of fit *S* are based on *F*^2^, conventional *R*-factors *R* are based on *F*, with *F* set to zero for negative *F*^2^. The threshold expression of *F*^2^ > σ(*F*^2^) is used only for calculating *R*-factors(gt) etc. and is not relevant to the choice of reflections for refinement. *R*-factors based on *F*^2^ are statistically about twice as large as those based on *F*, and *R*- factors based on ALL data will be even larger.

### Fractional atomic coordinates and isotropic or equivalent isotropic displacement parameters (Å^2^)

**Table:**

	*x*	*y*	*z*	*U*_iso_*/*U*_eq_	Occ. (<1)
Ni1	0.254825 (15)	0.506019 (16)	0.523912 (16)	0.01040 (6)	
P1	0.39949 (3)	0.31363 (3)	0.57666 (3)	0.01121 (10)	
P2	0.37259 (3)	0.41840 (3)	0.35969 (3)	0.01008 (9)	
P3	0.06502 (3)	0.41352 (3)	0.36142 (3)	0.01021 (9)	
P4	0.09045 (3)	0.64384 (3)	0.36384 (3)	0.01202 (10)	
C1	0.36922 (12)	0.29690 (12)	0.42905 (13)	0.0115 (3)	
C2	0.43105 (13)	0.21769 (14)	0.39811 (14)	0.0168 (4)	
H1C	0.4243	0.1528	0.4319	0.025*	
H2C	0.4953	0.2400	0.4229	0.025*	
H3C	0.4132	0.2096	0.3197	0.025*	
C3	0.06373 (12)	0.52864 (13)	0.28050 (13)	0.0119 (3)	
C4	−0.02889 (13)	0.53922 (15)	0.19427 (14)	0.0179 (4)	
H4C	−0.0284	0.6008	0.1520	0.027*	
H5C	−0.0785	0.5436	0.2291	0.027*	
H6C	−0.0388	0.4801	0.1466	0.027*	
N1	0.27160 (11)	0.25917 (12)	0.39567 (12)	0.0126 (3)	
H1N	0.2545 (15)	0.2521 (17)	0.3304 (19)	0.019*	
H2N	0.2367 (16)	0.2994 (18)	0.4148 (18)	0.019*	
H3N	0.2685 (15)	0.2013 (18)	0.4245 (17)	0.019*	
N2	0.13780 (11)	0.51860 (12)	0.22423 (12)	0.0146 (3)	
H4N	0.1273 (15)	0.4615 (18)	0.1846 (18)	0.022*	
H5N	0.1925 (17)	0.5163 (17)	0.269 (2)	0.022*	
H6N	0.1398 (15)	0.5704 (18)	0.1835 (18)	0.022*	
O1	0.32986 (8)	0.38339 (9)	0.60142 (9)	0.0134 (2)	
O2	0.40603 (9)	0.20946 (9)	0.62466 (9)	0.0160 (3)	
O3	0.49780 (9)	0.36086 (10)	0.60780 (10)	0.0170 (3)	
H3O	0.5017 (16)	0.4180 (18)	0.6090 (18)	0.020*	
O4	0.31223 (9)	0.49330 (9)	0.39539 (10)	0.0134 (3)	
O5	0.32244 (9)	0.39486 (10)	0.23893 (10)	0.0153 (3)	
H5O	0.3493 (16)	0.3661 (18)	0.2144 (19)	0.018*	
O6	0.47117 (8)	0.44797 (9)	0.37730 (10)	0.0159 (3)	
O7	0.15774 (8)	0.40580 (9)	0.44333 (9)	0.0129 (2)	
O8	0.05982 (9)	0.32515 (10)	0.28082 (10)	0.0168 (3)	
H8O	0.0131 (16)	0.2986 (18)	0.2607 (18)	0.020*	
O9	−0.01636 (8)	0.41774 (10)	0.40575 (10)	0.0156 (3)	
O10	0.18139 (9)	0.62792 (9)	0.44657 (9)	0.0152 (3)	
O11	0.08907 (9)	0.73164 (9)	0.28909 (10)	0.0166 (3)	
O12	0.00776 (9)	0.65624 (10)	0.41202 (11)	0.0176 (3)	
H12O	0.0136 (16)	0.6311 (18)	0.4639 (19)	0.021*	
O13	0.35399 (10)	0.60034 (11)	0.61432 (11)	0.0191 (3)	
H131	0.4115 (18)	0.5931 (17)	0.6123 (18)	0.023*	
H132	0.3421 (16)	0.6536 (19)	0.6076 (19)	0.023*	
O14	0.18989 (10)	0.51133 (10)	0.64265 (11)	0.0165 (3)	
H141	0.1422 (18)	0.5294 (18)	0.6272 (19)	0.020*	
H142	0.2110 (16)	0.5442 (17)	0.6965 (19)	0.020*	
O15	0.19397 (11)	0.29225 (11)	0.67872 (11)	0.0218 (3)	
H151	0.1539 (18)	0.3253 (19)	0.673 (2)	0.026*	
H152	0.2340 (17)	0.3238 (18)	0.6574 (19)	0.026*	
O16	0.10537 (12)	0.37339 (13)	0.06297 (12)	0.0288 (3)	
H161	0.1308 (18)	0.317 (2)	0.095 (2)	0.035*	
H162	0.1417 (19)	0.393 (2)	0.035 (2)	0.035*	
O17	0.24192 (11)	0.06113 (11)	0.46573 (12)	0.0243 (3)	
H171	0.2308 (18)	0.0144 (19)	0.423 (2)	0.029*	
H172	0.2931 (18)	0.0380 (19)	0.510 (2)	0.029*	
O18	0.22367 (11)	0.61233 (12)	0.83215 (12)	0.0263 (3)	
H181	0.2724 (18)	0.6606 (19)	0.8440 (19)	0.032*	
H182	0.1789 (18)	0.652 (2)	0.822 (2)	0.032*	
O19	0.55044 (11)	0.07361 (13)	0.64598 (13)	0.0291 (3)	
H191	0.5136 (19)	0.118 (2)	0.644 (2)	0.035*	
H192	0.5823 (18)	0.0687 (19)	0.711 (2)	0.035*	
O20	0.30635 (14)	0.79284 (13)	0.63636 (17)	0.0427 (4)	
H201	0.336 (2)	0.851 (2)	0.621 (2)	0.051*	
H202	0.332 (2)	0.803 (2)	0.707 (3)	0.051*	
O21	0.39921 (12)	−0.05323 (13)	0.55580 (15)	0.0333 (4)	
H211	0.447 (2)	−0.024 (2)	0.593 (2)	0.040*	
H212	0.4083 (19)	−0.069 (2)	0.493 (2)	0.040*	
O22A	0.67316 (15)	0.28256 (16)	0.6372 (3)	0.0332 (10)	0.863 (11)
H221	0.613 (2)	0.307 (2)	0.616 (2)	0.040*	
H222	0.7072 (19)	0.321 (2)	0.607 (2)	0.040*	
O23A	0.1136 (2)	0.6434 (4)	0.0445 (4)	0.0398 (14)	0.81 (2)
H231	0.068 (2)	0.671 (3)	0.025 (3)	0.048*	
H232	0.162 (2)	0.684 (2)	0.063 (2)	0.048*	
O22B	0.6571 (14)	0.2621 (15)	0.577 (2)	0.058 (6)*	0.137 (11)
O23B	0.1072 (11)	0.6784 (17)	0.0740 (16)	0.045 (5)*	0.19 (2)

### Atomic displacement parameters (Å^2^)

**Table:**

	*U*^11^	*U*^22^	*U*^33^	*U*^12^	*U*^13^	*U*^23^
Ni1	0.00890 (11)	0.01126 (11)	0.01095 (10)	0.00122 (8)	0.00259 (8)	−0.00011 (7)
P1	0.0109 (2)	0.0114 (2)	0.01128 (18)	0.00152 (16)	0.00294 (16)	0.00155 (15)
P2	0.0093 (2)	0.0103 (2)	0.01103 (18)	0.00031 (16)	0.00354 (15)	0.00037 (14)
P3	0.0084 (2)	0.0113 (2)	0.01100 (18)	−0.00023 (16)	0.00281 (15)	−0.00083 (15)
P4	0.0118 (2)	0.0113 (2)	0.01298 (19)	0.00264 (16)	0.00346 (16)	0.00198 (15)
C1	0.0107 (8)	0.0114 (8)	0.0126 (7)	0.0006 (6)	0.0035 (6)	0.0004 (6)
C2	0.0182 (9)	0.0159 (9)	0.0181 (8)	0.0056 (7)	0.0081 (7)	0.0008 (6)
C3	0.0098 (8)	0.0147 (8)	0.0120 (7)	0.0008 (6)	0.0043 (6)	0.0007 (6)
C4	0.0142 (9)	0.0230 (9)	0.0141 (8)	0.0009 (7)	0.0000 (7)	0.0028 (7)
N1	0.0126 (8)	0.0116 (7)	0.0140 (7)	−0.0005 (6)	0.0041 (6)	−0.0004 (6)
N2	0.0133 (8)	0.0173 (8)	0.0143 (7)	0.0009 (6)	0.0059 (6)	0.0019 (6)
O1	0.0137 (6)	0.0150 (6)	0.0122 (5)	0.0035 (5)	0.0045 (5)	0.0013 (4)
O2	0.0192 (7)	0.0135 (6)	0.0161 (6)	0.0021 (5)	0.0062 (5)	0.0039 (5)
O3	0.0121 (6)	0.0152 (6)	0.0219 (6)	−0.0008 (5)	0.0016 (5)	0.0000 (5)
O4	0.0146 (6)	0.0119 (6)	0.0157 (6)	0.0023 (5)	0.0076 (5)	0.0011 (4)
O5	0.0159 (7)	0.0181 (7)	0.0121 (6)	0.0019 (5)	0.0043 (5)	−0.0010 (5)
O6	0.0109 (6)	0.0162 (6)	0.0216 (6)	−0.0006 (5)	0.0063 (5)	0.0002 (5)
O7	0.0108 (6)	0.0111 (6)	0.0152 (6)	0.0000 (5)	0.0008 (5)	0.0004 (4)
O8	0.0140 (7)	0.0168 (6)	0.0201 (6)	−0.0043 (5)	0.0056 (5)	−0.0069 (5)
O9	0.0106 (6)	0.0205 (6)	0.0175 (6)	0.0024 (5)	0.0067 (5)	0.0021 (5)
O10	0.0153 (6)	0.0120 (6)	0.0163 (6)	0.0010 (5)	0.0010 (5)	0.0004 (4)
O11	0.0160 (7)	0.0148 (6)	0.0191 (6)	0.0028 (5)	0.0053 (5)	0.0064 (5)
O12	0.0185 (7)	0.0195 (7)	0.0175 (6)	0.0064 (5)	0.0095 (5)	0.0050 (5)
O13	0.0123 (7)	0.0156 (6)	0.0281 (7)	0.0001 (5)	0.0035 (5)	−0.0060 (5)
O14	0.0121 (7)	0.0230 (7)	0.0145 (6)	0.0048 (5)	0.0035 (5)	−0.0009 (5)
O15	0.0212 (8)	0.0214 (7)	0.0235 (7)	0.0042 (6)	0.0076 (6)	0.0070 (5)
O16	0.0346 (9)	0.0312 (8)	0.0223 (7)	0.0067 (7)	0.0107 (6)	0.0034 (6)
O17	0.0247 (8)	0.0186 (7)	0.0265 (7)	−0.0006 (6)	0.0018 (6)	0.0005 (6)
O18	0.0244 (8)	0.0219 (7)	0.0317 (8)	0.0003 (6)	0.0061 (6)	−0.0026 (6)
O19	0.0262 (9)	0.0312 (9)	0.0286 (8)	0.0084 (7)	0.0058 (7)	−0.0019 (6)
O20	0.0493 (12)	0.0245 (9)	0.0553 (11)	−0.0003 (8)	0.0161 (9)	−0.0062 (8)
O21	0.0259 (9)	0.0301 (9)	0.0413 (9)	0.0024 (7)	0.0051 (7)	−0.0056 (7)
O22A	0.0208 (12)	0.0242 (11)	0.058 (2)	0.0042 (8)	0.0156 (10)	0.0154 (10)
O23A	0.0491 (18)	0.038 (2)	0.0339 (18)	0.0057 (12)	0.0146 (12)	0.0157 (15)

### Geometric parameters (Å, °)

**Table:**

Ni1—O1	2.0689 (12)	N1—H3N	0.86 (2)
Ni1—O4	2.0861 (13)	N2—H4N	0.90 (2)
Ni1—O7	2.0355 (12)	N2—H5N	0.87 (2)
Ni1—O10	2.0508 (12)	N2—H6N	0.87 (2)
Ni1—O13	2.0477 (13)	O3—H3O	0.76 (2)
Ni1—O14	2.0424 (14)	O5—H5O	0.69 (2)
P1—O1	1.4999 (13)	O8—H8O	0.77 (2)
P1—O2	1.5003 (12)	O12—H12O	0.73 (2)
P1—O3	1.5596 (14)	O13—H131	0.88 (3)
P1—C1	1.8484 (16)	O13—H132	0.72 (2)
P2—O6	1.4976 (13)	O14—H141	0.73 (2)
P2—O4	1.5035 (13)	O14—H142	0.81 (2)
P2—O5	1.5660 (13)	O15—H151	0.73 (3)
P2—C1	1.8449 (17)	O15—H152	0.84 (3)
P3—O9	1.4978 (13)	O16—H161	0.88 (3)
P3—O7	1.5085 (12)	O16—H162	0.78 (3)
P3—O8	1.5515 (13)	O17—H171	0.81 (3)
P3—C3	1.8419 (17)	O17—H172	0.88 (3)
P4—O10	1.5048 (12)	O18—H181	0.95 (3)
P4—O11	1.5056 (12)	O18—H182	0.83 (3)
P4—O12	1.5547 (14)	O19—H191	0.81 (3)
P4—C3	1.8423 (17)	O19—H192	0.85 (3)
C1—N1	1.505 (2)	O20—H201	0.94 (3)
C1—C2	1.529 (2)	O20—H202	0.90 (3)
C2—H1C	0.9800	O21—H211	0.85 (3)
C2—H2C	0.9800	O21—H212	0.88 (3)
C2—H3C	0.9800	O22A—H221	0.94 (3)
C3—N2	1.503 (2)	O22A—H222	0.89 (3)
C3—C4	1.536 (2)	O23A—H231	0.76 (3)
C4—H4C	0.9800	O23A—H232	0.89 (3)
C4—H5C	0.9800	O22B—H221	1.12 (3)
C4—H6C	0.9800	O22B—H222	1.08 (3)
N1—H1N	0.82 (2)	O23B—H231	0.75 (3)
N1—H2N	0.83 (2)	O23B—H232	0.88 (3)
			
O1—Ni1—O4	91.65 (5)	H2C—C2—H3C	109.5
O7—Ni1—O10	92.51 (5)	N2—C3—C4	108.05 (14)
O14—Ni1—O13	88.83 (6)	N2—C3—P3	108.13 (11)
O7—Ni1—O14	88.69 (5)	C4—C3—P3	110.53 (12)
O7—Ni1—O13	175.95 (5)	N2—C3—P4	106.51 (12)
O14—Ni1—O10	91.76 (5)	C4—C3—P4	110.93 (12)
O13—Ni1—O10	90.77 (5)	P3—C3—P4	112.48 (9)
O7—Ni1—O1	87.74 (5)	C3—C4—H4C	109.5
O14—Ni1—O1	88.67 (5)	C3—C4—H5C	109.5
O13—Ni1—O1	88.99 (5)	H4C—C4—H5C	109.5
O10—Ni1—O1	179.50 (5)	C3—C4—H6C	109.5
O7—Ni1—O4	86.64 (5)	H4C—C4—H6C	109.5
O14—Ni1—O4	175.30 (5)	H5C—C4—H6C	109.5
O13—Ni1—O4	95.86 (5)	C1—N1—H1N	109.7 (16)
O10—Ni1—O4	87.94 (5)	C1—N1—H2N	110.9 (15)
O1—P1—O2	116.32 (7)	H1N—N1—H2N	110 (2)
O1—P1—O3	112.12 (7)	C1—N1—H3N	109.8 (14)
O2—P1—O3	108.12 (7)	H1N—N1—H3N	108 (2)
O1—P1—C1	107.88 (7)	H2N—N1—H3N	109 (2)
O2—P1—C1	106.67 (7)	C3—N2—H4N	108.2 (15)
O3—P1—C1	105.00 (8)	C3—N2—H5N	112.4 (16)
O6—P2—O4	116.57 (7)	H4N—N2—H5N	109 (2)
O6—P2—O5	112.91 (8)	C3—N2—H6N	112.4 (15)
O4—P2—O5	105.62 (7)	H4N—N2—H6N	110 (2)
O6—P2—C1	108.30 (8)	H5N—N2—H6N	104 (2)
O4—P2—C1	108.70 (8)	P1—O1—Ni1	134.46 (7)
O5—P2—C1	103.96 (7)	P1—O3—H3O	117.9 (18)
O9—P3—O7	115.95 (7)	P2—O4—Ni1	136.17 (7)
O9—P3—O8	113.07 (8)	P2—O5—H5O	112.4 (19)
O7—P3—O8	106.51 (7)	P3—O7—Ni1	135.55 (7)
O9—P3—C3	107.89 (8)	P3—O8—H8O	116.2 (18)
O7—P3—C3	108.38 (7)	P4—O10—Ni1	136.12 (8)
O8—P3—C3	104.34 (7)	P4—O12—H12O	114.5 (19)
O10—P4—O11	114.22 (7)	Ni1—O13—H131	119.3 (15)
O10—P4—O12	114.15 (7)	Ni1—O13—H132	113.7 (19)
O11—P4—O12	107.92 (7)	H131—O13—H132	108 (2)
O10—P4—C3	108.02 (7)	Ni1—O14—H141	116.7 (19)
O11—P4—C3	107.33 (7)	Ni1—O14—H142	120.6 (17)
O12—P4—C3	104.55 (8)	H141—O14—H142	101 (2)
N1—C1—C2	108.83 (14)	H151—O15—H152	109 (3)
N1—C1—P2	107.83 (11)	H161—O16—H162	102 (3)
C2—C1—P2	111.41 (12)	H171—O17—H172	99 (2)
N1—C1—P1	106.09 (11)	H181—O18—H182	100 (2)
C2—C1—P1	110.99 (11)	H191—O19—H192	106 (3)
P2—C1—P1	111.47 (9)	H201—O20—H202	90 (2)
C1—C2—H1C	109.5	H211—O21—H212	108 (3)
C1—C2—H2C	109.5	H221—O22A—H222	108 (2)
H1C—C2—H2C	109.5	H231—O23A—H232	115 (3)
C1—C2—H3C	109.5	H221—O22B—H222	85 (3)
H1C—C2—H3C	109.5	H231—O23B—H232	117 (4)
			
O6—P2—C1—N1	−170.58 (11)	O12—P4—C3—C4	57.74 (14)
O4—P2—C1—N1	61.89 (12)	O10—P4—C3—P3	55.30 (11)
O5—P2—C1—N1	−50.27 (13)	O11—P4—C3—P3	178.91 (9)
O6—P2—C1—C2	−51.23 (13)	O12—P4—C3—P3	−66.64 (10)
O4—P2—C1—C2	−178.76 (11)	O2—P1—O1—Ni1	−153.15 (9)
O5—P2—C1—C2	69.08 (13)	O3—P1—O1—Ni1	81.70 (11)
O6—P2—C1—P1	73.36 (10)	C1—P1—O1—Ni1	−33.43 (12)
O4—P2—C1—P1	−54.17 (11)	O7—Ni1—O1—P1	87.56 (11)
O5—P2—C1—P1	−166.33 (9)	O14—Ni1—O1—P1	176.31 (11)
O1—P1—C1—N1	−55.84 (12)	O13—Ni1—O1—P1	−94.84 (11)
O2—P1—C1—N1	69.81 (12)	O4—Ni1—O1—P1	1.00 (11)
O3—P1—C1—N1	−175.58 (11)	O6—P2—O4—Ni1	−104.40 (11)
O1—P1—C1—C2	−173.91 (12)	O5—P2—O4—Ni1	129.32 (11)
O2—P1—C1—C2	−48.25 (14)	C1—P2—O4—Ni1	18.27 (13)
O3—P1—C1—C2	66.35 (14)	O7—Ni1—O4—P2	−79.32 (11)
O1—P1—C1—P2	61.27 (11)	O13—Ni1—O4—P2	97.48 (11)
O2—P1—C1—P2	−173.07 (8)	O10—Ni1—O4—P2	−171.96 (11)
O3—P1—C1—P2	−58.47 (11)	O1—Ni1—O4—P2	8.33 (11)
O9—P3—C3—N2	−172.77 (11)	O9—P3—O7—Ni1	−91.92 (12)
O7—P3—C3—N2	60.95 (12)	O8—P3—O7—Ni1	141.28 (10)
O8—P3—C3—N2	−52.26 (12)	C3—P3—O7—Ni1	29.52 (13)
O9—P3—C3—C4	−54.68 (14)	O14—Ni1—O7—P3	89.45 (11)
O7—P3—C3—C4	179.03 (12)	O10—Ni1—O7—P3	−2.25 (11)
O8—P3—C3—C4	65.82 (14)	O1—Ni1—O7—P3	178.18 (11)
O9—P3—C3—P4	69.91 (10)	O4—Ni1—O7—P3	−90.04 (11)
O7—P3—C3—P4	−56.38 (11)	O11—P4—O10—Ni1	−146.97 (10)
O8—P3—C3—P4	−169.59 (9)	O12—P4—O10—Ni1	88.17 (12)
O10—P4—C3—N2	−62.98 (12)	C3—P4—O10—Ni1	−27.64 (13)
O11—P4—C3—N2	60.62 (12)	O7—Ni1—O10—P4	1.17 (12)
O12—P4—C3—N2	175.08 (10)	O14—Ni1—O10—P4	−87.60 (12)
O10—P4—C3—C4	179.67 (12)	O13—Ni1—O10—P4	−176.46 (12)
O11—P4—C3—C4	−56.72 (14)	O4—Ni1—O10—P4	87.71 (12)

### Hydrogen-bond geometry (Å, °)

**Table:**

*D*—H···*A*	*D*—H	H···*A*	*D*···*A*	*D*—H···*A*
N1—H1N···O15^i^	0.82 (2)	2.01 (2)	2.807 (2)	166 (2)
N1—H2N···O7	0.83 (2)	1.95 (2)	2.773 (2)	172 (2)
N1—H3N···O17	0.86 (2)	2.00 (2)	2.843 (2)	169 (2)
N2—H4N···O16	0.90 (2)	1.91 (2)	2.774 (2)	160 (2)
N2—H5N···O4	0.87 (2)	2.10 (3)	2.955 (2)	169 (2)
N2—H6N···O23A	0.87 (2)	1.98 (2)	2.789 (3)	154 (2)
O3—H3O···O6^ii^	0.76 (2)	1.81 (2)	2.5637 (18)	172 (2)
O5—H5O···O2^i^	0.69 (2)	1.91 (2)	2.5930 (18)	170 (3)
O8—H8O···O11^iii^	0.77 (2)	1.74 (2)	2.5075 (18)	176 (3)
O12—H12O···O9^iv^	0.73 (2)	1.79 (2)	2.5209 (18)	172 (3)
O13—H131···O6^ii^	0.88 (3)	1.83 (3)	2.696 (2)	167 (2)
O13—H132···O20	0.72 (2)	1.98 (3)	2.678 (2)	162 (3)
O14—H141···O9^iv^	0.73 (2)	1.96 (3)	2.6936 (19)	176 (3)
O14—H142···O18	0.81 (2)	1.93 (2)	2.711 (2)	162 (2)
O15—H151···O12^iv^	0.73 (3)	2.40 (3)	3.029 (2)	145 (2)
O15—H152···O1	0.84 (3)	1.96 (3)	2.797 (2)	174 (2)
O16—H161···O15^i^	0.88 (3)	1.90 (3)	2.775 (2)	172 (2)
O16—H162···O17^i^	0.78 (3)	2.06 (3)	2.838 (2)	177 (3)
O17—H171···O18^i^	0.81 (3)	2.03 (3)	2.835 (2)	170 (3)
O17—H172···O21	0.88 (3)	1.96 (3)	2.786 (2)	155 (2)
O18—H181···O22A^v^	0.95 (3)	1.79 (3)	2.702 (2)	158 (2)
O18—H182···O11^vi^	0.83 (3)	2.02 (3)	2.841 (2)	168 (2)
O19—H191···O2	0.81 (3)	1.98 (3)	2.781 (2)	169 (3)
O19—H192···O13^vii^	0.85 (3)	2.23 (3)	3.055 (2)	163 (2)
O20—H201···O21^viii^	0.94 (3)	1.91 (3)	2.829 (3)	164 (3)
O20—H202···O22A^v^	0.90 (3)	2.05 (3)	2.861 (4)	149 (3)
O21—H211···O19	0.85 (3)	1.99 (3)	2.814 (2)	163 (3)
O21—H212···O19^ix^	0.88 (3)	2.06 (3)	2.928 (3)	166 (3)
O22A—H221···O3	0.94 (3)	1.86 (3)	2.775 (2)	167 (3)
O22A—H222···O10^ii^	0.89 (3)	2.10 (3)	2.958 (3)	161 (2)
O23A—H231···O16^x^	0.76 (3)	2.62 (3)	3.225 (4)	139 (3)
O23A—H232···O20^xi^	0.89 (3)	2.14 (3)	2.951 (4)	150 (3)

Symmetry codes: (i) *x*, −*y*+1/2, *z*−1/2; (ii) −*x*+1, −*y*+1, −*z*+1; (iii) −*x*, *y*−1/2, −*z*+1/2; (iv) −*x*, −*y*+1, −*z*+1; (v) −*x*+1, *y*+1/2, −*z*+3/2; (vi) *x*, −*y*+3/2, *z*+1/2; (vii) −*x*+1, *y*−1/2, −*z*+3/2; (viii) *x*, *y*+1, *z*; (ix) −*x*+1, −*y*, −*z*+1; (x) −*x*, −*y*+1, −*z*; (xi) *x*, −*y*+3/2, *z*−1/2.
